# Spinal Dural Arteriovenous Fistula: Insights Into Operative Management

**DOI:** 10.7759/cureus.38448

**Published:** 2023-05-02

**Authors:** Ali Msheik, Zeinab Al Mokdad, Teddy Gerges, Ahmad Aoude

**Affiliations:** 1 Neurological Surgery, Lebanese University Faculty of Medical Sciences, Hadath, LBN; 2 Public Health, Lebanese University Faculty of Medical Sciences, Hadath, LBN; 3 Anesthesiology, Winchester Anesthesia Associates Inc., Winchester, USA; 4 Neurological Surgery, Al Rassoul Al Azam Hospital, Beirut, LBN

**Keywords:** weakness, spinal venous congestion, urinary retention, erectile dysfunction, sdavf

## Abstract

Spinal Dural Arteriovenous Fistula (SDAVF) is a rare and complex vascular condition with significant neurological consequences if left untreated. We present a case of SDAVF in a 46-year-old male who presented with progressive myelopathy. The patient presented with a three-month history of progressive lower extremity weakness, numbness, urinary retention, constipation, and gait disturbance. The spine's magnetic resonance imaging (MRI) showed diffuse T2 hyperintensity and contrast enhancement from T11 to L1, raising the suspicion of an intradural spinal cord lesion. Further evaluation with spinal angiography revealed an SDAVF at the level of T11-T12. The patient underwent surgical resection of the fistula, His lower extremity weakness and numbness improved significantly after surgery, and he was discharged with a plan for close follow-up. Early diagnosis and appropriate treatment prevent neurological deficits and improve patient outcomes. Surgical resection of the fistula can significantly improve neurological symptoms and should be considered a treatment option for SDAVF.

## Introduction

Spinal dural arteriovenous fistulas (SDAVFs) are abnormal connections between arteries and veins within the dura mater of the spinal cord [1]. These rare vascular malformations account for approximately 70-80% of all spinal vascular lesions [2]. SDAVFs are most commonly diagnosed in males 50-70 years old [3]. The incidence of SDAVFs is estimated to be 5-10 cases per million people per year [4]. The pathophysiology of SDAVFs involves the formation of a direct arteriovenous shunt within the spinal dura mater [5]. This leads to venous hypertension, which can cause spinal cord ischemia and myelopathy [6]. Symptoms of SDAVFs can vary depending on the location and severity of the lesion. The most common symptoms include progressive myelopathy, lower extremity weakness, sensory disturbance, and bowel/bladder dysfunction [7]. Risk factors for SDAVFs include male sex, older age, hypertension, and a history of spinal trauma or surgery [8]. Diagnosis of SDAVFs typically involves magnetic resonance imaging (MRI) and spinal angiography [9]. Spinal angiography is considered the gold standard for diagnosis and allows for visualization of the fistula and its blood supply. Treatment options for SDAVFs include endovascular embolization, microsurgical disconnection, and stereotactic radiosurgery [10]. The choice of treatment depends on the location, size, and complexity of the lesion, as well as the patient's overall health status. The prognosis for SDAVFs depends on the lesion's severity and the treatment's timeliness. With early diagnosis and appropriate treatment, many patients experience significant improvement in symptoms and quality of life [11].

## Case presentation

We present the case of a 43-years old male patient who presented to the general practice (GP) clinics for lower back pain, urinary retention, constipation, and erectile dysfunction. The patient reported noticing mild lower limb pain on exertion since December 2022. The progression of the symptoms was insidious. As lower limb weakness worsened, the patient reported repetitive episodes of constipation and lowered voiding volume. Erectile dysfunction was the last symptom to develop. The patient presented to the GP clinic on February 16, 2022. Upon assessment, the patient showed no signs of systemic illness. Bilaterally, the sensation was preserved in the lower limbs except for saddle insensitivity. However, the motor power was 2/5. The patient failed to ambulate independently and reported pain upon movement of more than 10 meters. Of note, both ankle and knee reflexes were weak. At this stage, a non-exhaustive list of differential diagnoses included conus medullary syndrome, cauda equina syndrome, spinal cord tumor, and acute demyelinating polyradiculopathy. 

**Figure 1:**
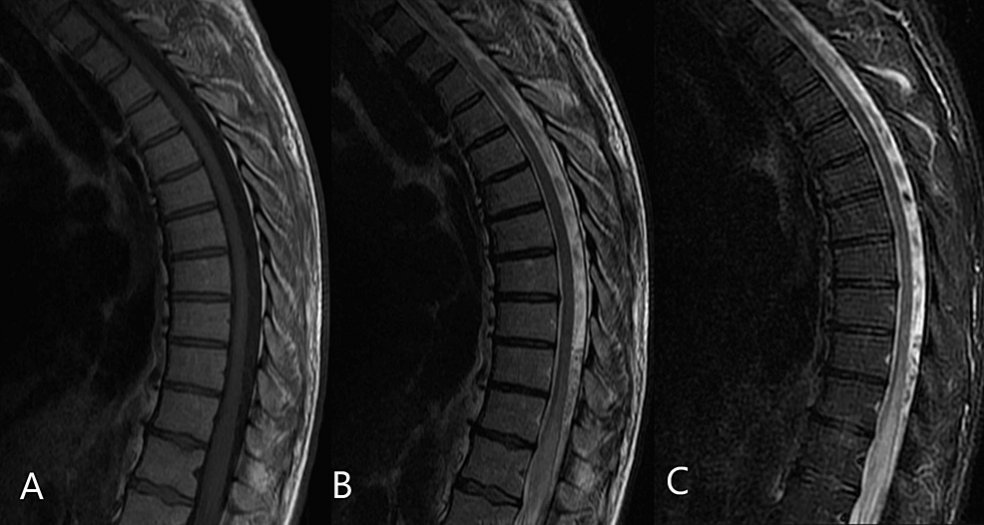
Sagittal cut of the lumbar spine of the patient. A: T1 sequence; B: T2 sequence; C: STIR sequence

**Figure 2:**
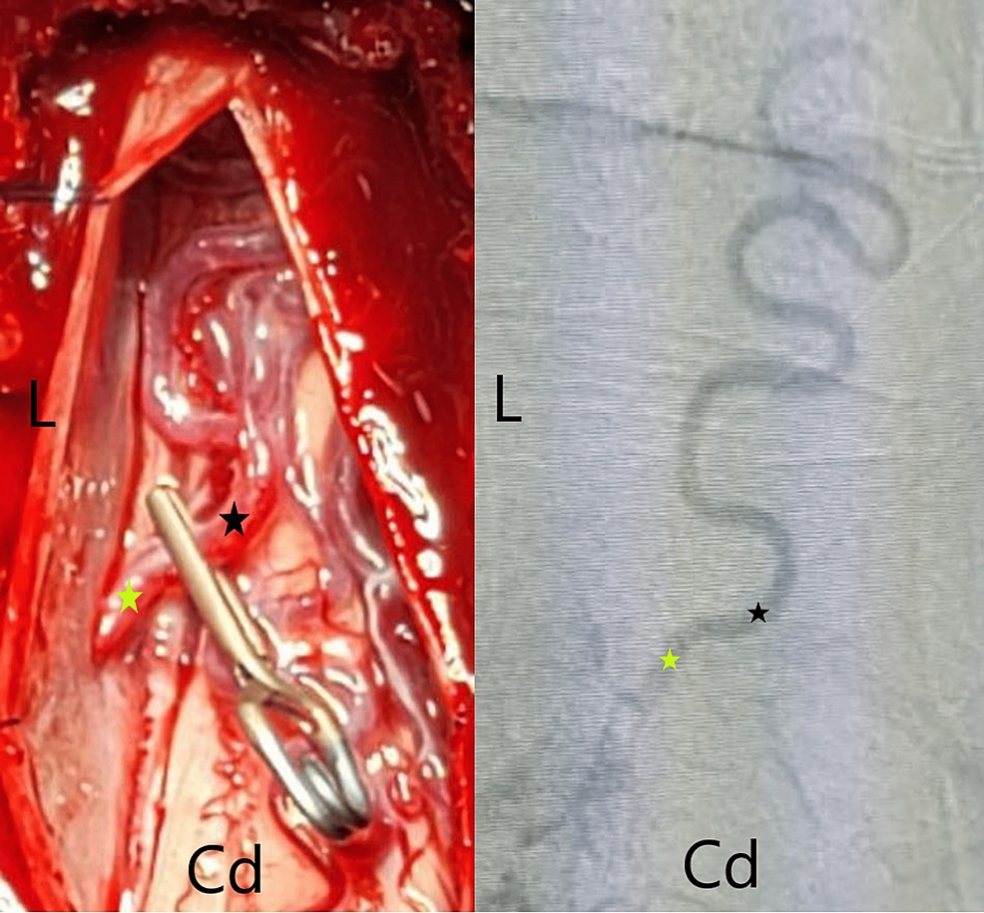
The analogy between the Digital Subtraction Angiography (DSA) (right side) and the surgical findings (left side) Cd: caudal side of the patient, L: left side of the patient, black star: venous part of the SDAVF, yellow star: Dural perforating artery. SDAVF: Spinal Dural Arteriovenous Fistula

Laboratory workup showed normal results except for mildly lowered serum potassium levels. This was attributed to the decreased per os intake reported by the patient due to constipation and dependency on laxatives. A Magnetic resonance imaging (MRI) of the dorsal and lumbar spine revealed intramedullary hypointensity on T1 sagittal sequence (Figure 1A) and flow voids on the cord surface on T2 sagittal sequence (Figure 1A). It diffused multilevel intramedullary hyperintensity (edema) on T2 sagittal sequence (Figures 1B, 1C). The patient was referred to the neurological surgery clinic, and an SDAVF was suspected. A digital subtraction angiography (DSA) of the brain was done on March 4, 2022, and a diagnosis of an SDVAF at T11-T12 vertebral levels was definite (figure 2).

The attending physician clarified the situation and the diagnosis of SDAVF for the patient. Although the patient was offered endovascular and surgical intervention modalities, he insisted on surgical intervention due to his condition's higher associated definitive treatment and curability. Informed consent was taken, and the patient was scheduled for surgery two weeks after the diagnosis. 

**Figure 3:**
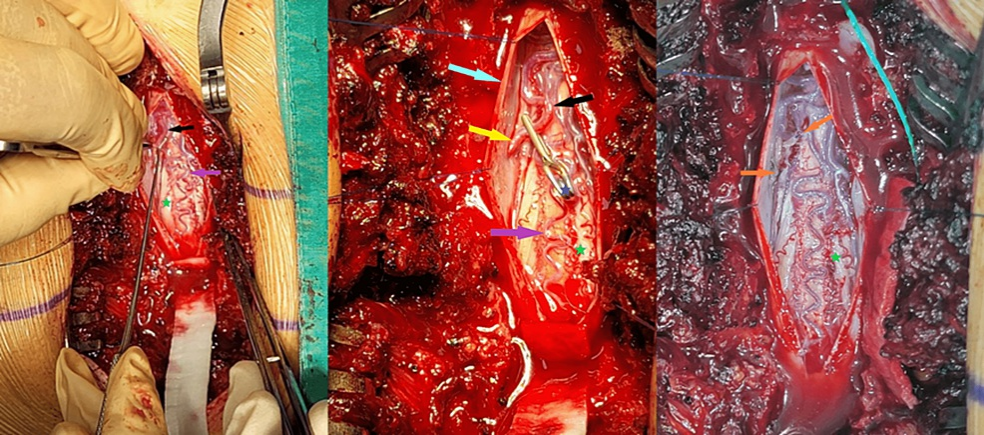
Surgical findings with annotation. Arrows by color. Yellow: Perforating dural artery, black: venous part of SDAVF, Purple: congested spinal vein, Orange: SDAVF ends after resection. Blue star: Vascular clip, Green star: decongested spinal vein after SDAVF resection. SDAVF: Spinal Dural Arteriovenous Fistula

Open surgical resection of the SDAVF was done on March 20, 2022 (Figure 2, 3). A longitudinal 10 cm incision was made along the midline in a prone position along the T10-L1 vertebral levels after localization by x-ray. Dissection was done bilaterally until the identification of the bony anatomy. Laminectomy of the T11-T12 vertebrae was done, and the dura was exposed. A longitudinal incision was made along the dura, and the SDAVF was identified. Ligation and resection of the fistula were done. The dura was closed in a watertight fashion using Prolene 5 sutures. The incision was closed layer by layer.

**Figure 4:**
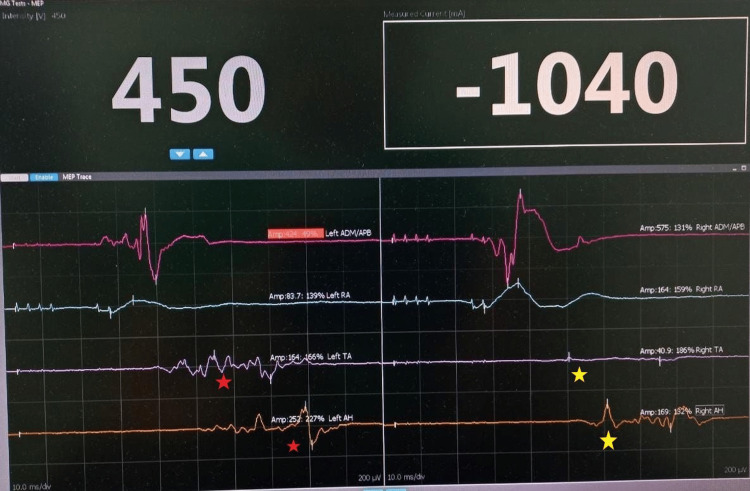
Recordings of neural electric conduction before the resection of the SDAVF. Red star: low voltage; Yellow star: Low current conduction. SDAVF: Spinal Dural Arteriovenous Fistula

**Figure 5:**
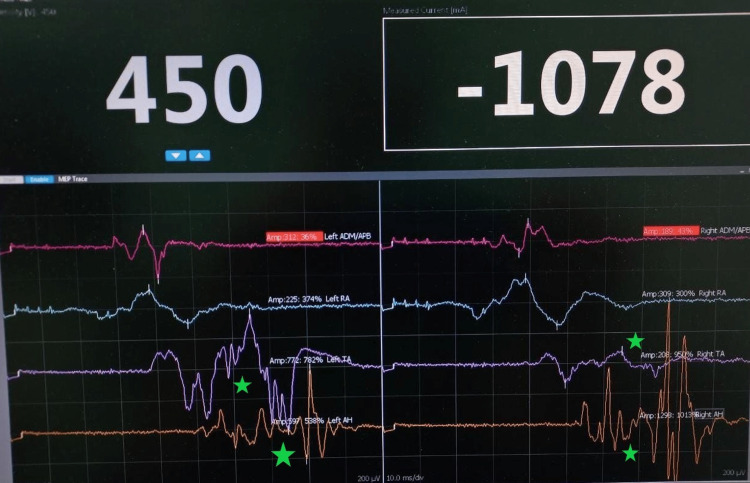
Recordings of neural electric conduction after the resection of the SDAVF. Green star: 4x-7x increase in the voltage and current recordings after the resection. SDAVF: Spinal Dural Arteriovenous Fistula

During the procedure, monitoring of the nerve conductivity was present. A comparison of the results before and after the resection of the SDAVF showed increased conductivity (Figures 4, 5).

After the procedure, the patient was examined on day one. He reported improved ambulation, clear by his ability to walk unassisted. Motor power increased to 4/5 through day two. Physiotherapy and rehabilitation therapy for his gastrointestinal and genitourinary dysfunction was advised, and the patient was discharged on day two with no complications.

## Discussion

Spinal dural arteriovenous fistulas (SDAVFs) are abnormal connections between arteries and veins in the spinal cord's protective covering. This condition can lead to progressive neurological deficits if left untreated. Currently, two main treatment modalities are used for SDAVFs: endovascular treatment and surgical treatment [12, 13]. Endovascular treatment involves using catheters and embolic agents to close off the abnormal connection between arteries and veins. This approach is less invasive than surgical treatment, and patients typically have a shorter hospital stay and recovery time. Endovascular treatment is also associated with a lower risk of complications such as bleeding, infection, and nerve damage. Additionally, endovascular treatment can be performed on unsuitable surgical candidates, such as those with multiple medical comorbidities or advanced age [12].

On the other hand, surgical treatment involves completely removing the abnormal connection between arteries and veins in the spinal cord's protective covering. This approach requires a more invasive procedure, which can lead to a longer hospital stay and recovery time. However, surgical treatment is considered more effective than endovascular treatment in completely curing SDAVF, with success rates of around 90%. In contrast, endovascular treatment has a success rate of approximately 60-70%. Surgical treatment is also preferred for patients with complex SDAVFs, which may not be amenable to endovascular treatment [13].

Both endovascular and surgical treatment of SDAVF has their advantages and disadvantages. Endovascular treatment is less invasive, associated with a lower risk of complications, and can be performed on unsuitable surgical candidates. However, surgical treatment is considered more effective in completely curing SDAVF and is preferred for complex SDAVFs. Ultimately, the choice of treatment modality should be based on the patient's characteristics and the specific features of their SDAVF. As for this patient, the explanation of both procedures and the effectiveness of each and the related complications and given that the patient is a good surgical candidate, surgical resection of the SDAVF was preferred.

Neural conductivity monitoring is a technique used to evaluate the function of the spinal cord during surgery. The use of neural conductivity monitoring during SDAVF surgery is effective in reducing the risk of neurological deficits. A study conducted in 2018 found that intraoperative neurophysiological monitoring during SDAVF surgery significantly reduced the incidence of new neurological deficits postoperatively [14]. Another study by Alzhrani et al. (2020) also reported that neural conductivity monitoring during SDAVF surgery was associated with a lower incidence of postoperative neurological deficits [15].

Neural conductivity monitoring is an essential technique during SDAVF surgery to prevent injury to the spinal cord. The use of this technique is effective in reducing the risk of neurological deficits postoperatively. Therefore, it is recommended that neural conductivity monitoring be performed during SDAVF surgery. For this patient, neural conductivity monitoring showed increased values after the resection of the SDAVF, which correlates with the clinical findings a few days after the procedure.

## Conclusions

The presented case report highlights the successful surgical treatment of a patient with SDAVF. The patient experienced significant improvement in symptoms following the procedure and did not experience any complications during or after surgery. The case report underscores the importance of early diagnosis and appropriate treatment of SDAVF to prevent neurological deficits and improve patient outcomes. SDAVF is a rare and complex vascular condition with significant neurological consequences if left untreated. While the exact cause of SDAVF is unknown, several risk factors have been identified. Early diagnosis and appropriate treatment prevent neurological deficits and improve patient outcomes. Further research is needed to understand better the epidemiology and pathophysiology of SDAVF, which can inform the development of more effective treatment strategies.
